# A New Observation in Dentinal Structure

**Published:** 1884-05

**Authors:** Wm. C. Britton

**Affiliations:** Detroit, Mich.


### A New Observation in Dentinal Structure.

BY WM. C. BRITTON, DETROIT, MICH.

Some three years since, in an article in the *American Journal of Microscopy*, I described the "Lamellated Structure of Dentine,"

which I had then just discovered quite accidentally; since which time I have also brought the subject to the notice of a number of prominent members of the dental profession; but, notwithstanding the important bearing the existence of this peculiar feature of structure must have in relating the teeth to other tissues, it has not as yet, I believe, been either refuted or verified. Trusting that some reader of this journal may take interest enough in this subject to investigate it, I will give some of my methods of examination and their results.

From my own investigations I am fully assured that the teeth are possessed of a *true laminated structure*—that is, "composed of thin plates or scales" (Webster). These laminæ being superinposed one upon another concentric to the axis of the tooth from the pulp to the periphery.

These lamellæ are *very* thin, being thicker in the cement substance than in the "granular layer," and somewhat thicker in the dentine, diminishing in thickness as they approach the pulp. All of these characteristics are capable of very easy demonstration. In searching the literature I have not been able to find any mention made of this feature of structure; in fact, *no* allusion is made to it except by Stricker, who, in referring to the lines discribed by Owen & Kolleker, (contour lines) says: "We are not however justified from these appearences in concluding that the dentine possesses a lamellated structure." Salter says it is rare to find any lamellated arrangement of the matrix of the *cementum*. Klein says, "The interglobular substance of czermak is imperfectly calcified dentine, and is present in distinct and separate layers, more or less parallel to the surface of the tooth." But, mark you, he says nothing about lamination. Now, a thing may be formed in "*distinct and separate layers*" and yet possess no feature of lamination. The word strata would just as appropriately have covered all that Klein discribes. He does not say that these "layers" may be separated, or that he has ever seen them in such condition.

Furthermore, granting his interpretation—the two forms of dentine (granular and compact) must be identical, and we are, I think, fully justified in inferring that whatever structure exists in one must exist in the other with, perhaps, slight modification. It would also seem very strange that so careful an investigator as Klein should have so lightly passed over so important a feature, had he seen any thing even to lead him to suspect its existence. These "layers" which he describes are most probably identical with the "development lines" of Salter & Owen, and are usually seen in teeth of imperfect development requiring no reagent to bring them out—and are, I believe, as has been stated by Tomes, "caused by interrupted nutrition," and should be regarded as pathological.

METHODS.—Select a perfectly sound, fully developed tooth; (an adult canine is a good tooth for this experiment;) place in a 75 per cent solution of caustic potash, keeping it hot, but not boiling. After ten or fifteen minutes examine the surface with the point of a dissecting needle, and as soon as the *cement* becomes loose remove it and examine with the microscope. Immerse the tooth again, watching it closely, and removing the lamellæ as often as they can be detached, keeping on in this way until all have been removed down to the pulp canal.

Now try another tooth, one, if possible, that has a calcified pulp with calcification also of the inter-tubular substance, and you will find the result the same. You will observe upon examination with the microscope that these lamellæ do not come off in single plates, but many of them together. Wash out the potash and mount some of them in glycerine and examine, first with a one inch and then with a quarter inch focus down across the edges of some of the blocks where they happen to be broken off obliquely and you will be able to count the number of lamellæ in the block. Observe the extreme thinness of the single lamina, also their relative thickness in the cement, granular layer. and the dentine.

One trial, I think, will convince any one that *lamination* is a persistent and normal feature of tooth structure. I have examined hundreds of teeth, and as yet have never failed to find this characteristic when sought for, regardless of condition—young or old, fresh or dry, normal or otherwise.

